# The impact mechanism and empirical analysis of financial efficiency of science and technology empowering regional real economy growth

**DOI:** 10.1371/journal.pone.0307497

**Published:** 2024-09-13

**Authors:** Tao Zhang, Jie Tian

**Affiliations:** School of Economics, Hebei GEO University, Shijiazhuang, China; University of International Business & Economics, CHINA

## Abstract

With the aim of exploring the impact mechanism of scientific and technological financial efficiency on regional real economy growth in the context of ecological civilization construction, this study introduces environmental regulation as a mediating factor. By analyzing changes in science and financial efficiency of science and technology, we provide an effective basis for regional real economy development. To achieve this goal, we define concepts such as science and financial efficiency of science and technology and regional real economy, measure data from 2012 to 2021, analyze the impact of science and financial efficiency of science and technology on economic growth using intermediary models, test mediation effects with bootstrap methods, and identify significant differences between regions. It indicates that enhancing science and financial efficiency of sci-tech benefits China’s regional real economy growth, but there’s unbalanced development across regions. Additionally, environmental regulation serves as a crucial intermediary in the relationship between sci-tech finance and economic growth. There exist regional disparities in the mediation effects of environmental regulation, with eastern regions demonstrating stronger effects compared to central and western regions.

## Introduction

In terms of the new normal of China’s economic development, it is imperative to comprehensively grasp the scientific connotation of high-quality development in order to facilitate the transformation towards enhanced quality, efficiency, and power. This can be achieved through robust power reforms that drive changes in economic development quality and efficiency, as well as by fostering scientific and technological innovation to enhance output quality and efficiency. Scientific and technological innovation takes precedence in promoting economic development and facilitating high-quality industrial growth. It serves as the fundamental driving force behind achieving high-quality development while simultaneously bolstering overall innovative capabilities. The significance of scientific and technological innovation has garnered increased attention with more profound discussions taking place on this subject matter; it has become a topic of great interest for society at large. The latest Chinese government work report explicitly emphasizes the need to strengthen support for scientific and technological innovation—an aspect particularly crucial within China’s context where such innovation plays an irreplaceable role in advancing industrial economy, national economy, and regional real economy.

In the context of scientific and technological innovation, the deep integration of science, technology, and finance has facilitated the emergence of "scientific and technological finance". Scientific and technological finance serves as a fundamental component within both national innovation systems and financial systems. The government’s financial sector takes the lead in guiding numerous financial institutions and intermediary service agencies to continuously innovate financial products, establish corresponding service platforms, optimize service models, thereby promoting the development of the real economy. The efficiency of technological financing operations has always been an urgent issue that needs to be addressed–specifically how to maximize output from investments in scientific and technological achievements. This disparity between regions can impact the efficiency of science and technology finance output while diminishing its substantive promotion effect on regional real economies. Taking Shanghai as an example, the city has established 17 scientific and technological financial service stations, serving enterprises more than 2,000 times a year, effectively promoting the docking between the government, financial institutions, and scientific and technological innovation enterprises.

Consequently, it becomes evident that enabling regional entity growth through efficient sci-tech finance is crucial. The higher the input-output efficiency of sci-tech finance, the more significant its positive impact on regional economic growth. This study aims to investigate this positive relationship by examining existing mechanisms influencing it as well as exploring how science and technology finance empowers regional economic growth. Additionally, we introduce environmental regulation’s role in enabling such growth while laying a solid foundation for future developments within regional economies.

## Literature review

The research on technology finance can be traced back to the mid to late 20th century. Schumpeter clarified the advantages of the integration of finance and technology in his book “Economic Development Theory” and explained the supporting role of credit capital for technological innovation. Ang believes that technological innovation cannot do without the liberalization of financial development [1]. Peter also pointed out in his book “Technology Revolution and Financial Capital” that the new paradigm formed by the integration of technology and finance is more conducive to promoting the development of regional economy [2]. Guarnieri points out from the enterprise level that the integration between public financial investment and technological innovation promotes the development of technological innovation [3]. Similarly, Sasidharan also believes that the development of technology finance has played an important economic support role in enterprise product innovation [4]. Zetsche believes that the advantages of technology finance lie in improving business quality, improving risk management level, reducing transaction costs, and having good financial inclusivity, which can provide more credit support for the development of small and medium-sized enterprises and consumers in the regional real economy [5]. Guariglia’s research found that high-tech industries dominated by technological innovation are more susceptible to financial influences in the early stages of development, indicating that technology finance has a significant impact on local high-tech industries to a large extent [6]. Lenong’s discussion on the economic value and impact of technology finance indicates that technology finance is based on the integration of technology, innovation, and capital [7]. The vast majority will view “technology finance” as a result of the connection between technology and finance, which can have a positive impact on regional economy, industrial development, and personal credit. As Seoh believes, technology finance can promote national economic development on the one hand, and effectively improve the level of technological innovation on the other hand, thereby enhancing the competitiveness of national technology research and development [8]. At present, the academic community has conducted a detailed and in-depth analysis of technology finance, but has not provided a clear definition of it. Moreover, the mechanism of technology finance also needs further optimization.

Regarding the efficiency of technology finance, it generally refers to the efficiency of resource allocation in technology finance, especially under the role of innovation driven development strategies, the development scale of technology finance is increasingly expanding, and the efficiency of resource allocation in technology finance is more crucial due to limited financial resources [9]. The measurement and analysis of the efficiency of technology finance generally adopts frontier analysis, such as stochastic frontier production function, data envelopment analysis method, Malmquist index method and its improved model. After Charnes and Cooper proposed the DEA method, Banker made improvements to the DEA method and proposed the BBC model method [10]. Kundi used DEA models with constant and variable returns to scale to measure the efficiency of financial support. Huang used the DEA Malmquist index method to calculate the efficiency of technology finance, in order to analyze the differences in financial efficiency of science and technology between different regions [11]. Similarly, Yi reached similar conclusions using the DEA-BCC model and Gini coefficient analysis method [12].

By measuring the efficiency of technology finance, we have further analyzed the relationship between technology finance and regional real economic growth. As Rjoja and Valev pointed out in their research, the development of the financial industry has a strong stimulating effect on the real economy, and there are significant regional differences [13]. Adusei validated the relationship between financial development and economic growth in his research on economic and financial development in South Africa, indicating that the financial industry has a certain one-way promoting effect on economic development [14]. Peng analyzed the impact of environmental regulations on the efficiency of technology finance from empirical evidence from provincial-level administrative regions in China, and constructed a model to measure the efficiency of technology finance. Based on the CCR model, the BCC model was improved to achieve the measurement of financial efficiency of science and technology [15]. Based on the measurement of the efficiency of technology finance, the impact mechanism of technology finance on regional real economic growth can be effectively analyzed. For example, Adusei analyzed the economic and financial development of South Africa, and based on the Granger causality test method, verified the impact relationship between financial development and economic growth. The results showed that the development of the financial industry can promote regional economic growth [14]. Peng pointed out that the impact mechanism between technology finance and real economic growth is largely influenced by environmental regulations, which is closely related to the impact of environmental regulations on the efficiency of technology finance [16]. Wasi uses DEA method to measure energy efficiency, which can also be applied to measure the financial efficiency of science and technology [17–19]. In addition, the inline study used data envelopment analysis (DEA-SBM) to measure the energy efficiency of Chinese provinces from 2004 to 2017. Mann-Whitney U test was used to explore whether there are significant differences in energy efficiency levels between China’s energy security policy and energy conservation and emission reduction policy. Meta-frontier analysis was used to further investigate the regional heterogeneity of production technology gap in east, middle and west China, in which DEA-SBM was used to measure efficiency [20–25]. In general, there are abundant researches on the evaluation of financial efficiency of science and technology. However, the current researches still face some challenges and limitations. First of all, although there are various models and methods used to measure the financial efficiency of science and technology, each method has its specific applicable conditions and limitations, so it needs to be selected and adjusted according to the specific situation in practical application. The evaluation of financial efficiency of science and technology is a complex and important research field, which needs constant exploration and innovation. Future research should focus on the improvement of influencing factors, mechanism and evaluation methods of S&T finance efficiency, so as to provide strong support for promoting the healthy development of S&T finance in our country.

To sum up, the existing research has made some progress in exploring the mechanism of technological finance’s influence on regional economic growth, but there are still some deficiencies and gaps. First of all, although the measurement model of technical finance efficiency has been established, most studies are still focused on the macro level, and there are few discussions on the micro level of technical finance efficiency. Secondly, the existing research on the relationship between technical finance and regional economic growth often ignores the differences between regions. Although studies have mentioned the impact of environmental regulations on the relationship between technological finance and real economic growth, research in this area is still weak. This study presents a comprehensive overview of the implications of technology finance and enhances its theoretical framework from a deeper perspective, aiming to further enhance and optimize China’s technology finance service system, thereby contributing to the development of the regional real economy. The interconnection between the technology finance system and the regional real economy is highly significant. This study is based on empirical evidence from various regions in China, utilizing statistical data analysis to conduct a thorough examination of how technology finance influences the development of the regional real economy. It explores the impact of technological advancements and financial efficiency on real economic growth within an environmental regulatory context. Moreover, it proposes corresponding solutions and suggestions for addressing challenges encountered during this developmental process, with an ultimate goal to elevate the level of regional real economic development while providing valuable insights for research conducted in other provinces and cities.

## Empirical analysis

### Measurement of variables

#### Efficiency of technology finance

The financial efficiency of science and technology can be measured using the DEA model, which constructs a production frontier to evaluate the relative distance between each DMU and the frontier, thereby obtaining an efficiency value. The DEA model demonstrates robustness and flexibility, making it adaptable to different scales and types of datasets. Given the challenges in data collection and processing within the field of science and technology finance, selecting a DEA model that is more adaptable with relatively low data requirements is crucial for accurately evaluating financial efficiency of science and technology. To begin with, constructing an efficient index system for science and technology finance is necessary. This index system focuses on the input and output aspects of science and technology finance. Specifically, investments in science and technology finance encompass bank credit investment in science and technology, VC/PE capital investment, investment in science and technology capital markets, government financial investments in science and technology, as well as R&D personnel investments. As for outputs related to science and technology finance, they include scientific research papers published, valid patent numbers obtained, as well as industrial added value generated by science-technology enterprises.

How to choose the above indicators is because as an important means to promote the development of science and technology and economic growth, the measurement of its efficiency is very important. Through the application of DEA model, the efficiency of science and technology finance can be quantitatively analyzed, so as to better understand its operating mechanism and existing problems, further reveal the inherent laws and potential problems of science and financial efficiency of science and technology, and provide a scientific basis for optimizing the allocation of science and technology financial resources and improving the efficiency of science and technology finance. As shown in Table 1, it is the efficiency index system of science and technology finance.

**Table 1:** Efficiency index system of science and technology finance.

Primary indicators	Secondary indicators
Investment in technology and finance	Bank technology credit investment
	Technology risk investment amount (VC and PE capital investment)
	Investment in technology capital market funds
	Government funding for science and technology
	R&D personnel input
Technology and finance output	Number of scientific and technological papers
	Number of valid patents
	Industrial added value of technology enterprises

As illustrated in Table 1 above, venture capital and private equity fund investments are pivotal concepts within the contemporary financial market, playing a crucial role in fostering scientific and technological innovation, facilitating the growth of small and medium-sized enterprises, and driving economic expansion [26]. Venture capital represents a specialized form of capital operation that focuses on investing in nascent or expanding stage enterprises with the aim of attaining substantial returns. Typically targeting innovative small to medium-sized enterprises exhibiting high growth potential, venture capital investments often involve significant technical barriers and possess considerable market prospects [27]. Private equity encompasses an unpublicized approach for raising funds from a limited number of investors to invest in non-publicly listed companies through equity investment, aiming to capitalize on enterprise growth-induced appreciation.

There is a scarcity of data on technology credit in the relevant literature pertaining to indicators for technology finance investment. The financing of Chinese technology-based enterprises predominantly relies on indirect funding. Therefore, the year-end debt of innovative technology industries in each province is selected as the primary measure for assessing technology credit. The quantification methods and data sources for other indicators are presented in Table 2.

**Table 2:** Explanation and data source of technology financial efficiency indicators.

Indicators	Explanation of indicators	Data sources
Bank technology credit investment	Year-end liabilities of high-tech industries in various regions	China Torch Yearbook
Technology risk investment amount (VC and PE capital investment)	VC and VE capital investment amount	Wind database
Investment in technology capital market funds	Total financing amount for technology enterprises in various regions	Wind database
Government funding for science and technology	Science and technology support in general public budget expenditures in various regions	China Statistical Yearbook
R&D personnel input	Number of R&D personnel	China Science and Technology Statistical Yearbook
Number of scientific and technological papers	Number of scientific and technological papers included in CPCI-S/EI/SCI	CPCI-S/EI/SCI data retrieval obtained
Number of valid patents	Number of patents	China National Intellectual Property Administration
Industrial added value of technology enterprises	The output value of scientific research activities in technology enterprises	China Science and Technology Statistical Yearbook, China Torch Yearbook, Wind database

To address the efficiency issue of technology finance, it is imperative to establish a robust evaluation index system and methodologies for assessing the financial efficiency of science and technology. Therefore, this study has opted to employ the Data Envelopment Analysis (DEA) method for implementation. DEA is a mathematical programming approach that enables an assessment of input-output indicators to evaluate the performance of similar departments, units, or types [28]. This method can further evaluate multiple input and output decision units of the same department, unit, and type [29]. Simultaneously, the DEA method is a non-parametric statistical approach that treats each decision-making unit as an evaluated entity. It identifies relevant issues by evaluating other decision-making units and constructs appropriate data models to analyze relative efficiency, determine potential input-output combinations within the production frontier, measure the distance between each decision-making unit and the production frontier, assess the effectiveness of DEA for each unit, and ultimately derive an evaluation ranking. Generally speaking, within the model-defined production possibility set, it is required to either maintain inputs while increasing outputs or maintain outputs while reducing inputs. Previous research has extensively employed DEA models in various industries primarily for assessing relative effectiveness of multiple inputs and outputs in social and economic domains; particularly suitable for analyzing benefits and efficiency in cultural industries and government sectors.

This study considers different provinces in the technology and finance industry as different types of decision-making units. For each decision-making unit, there are m types of inputs and p types of outputs. Therefore, the input vector can be represented by Formula (1), and the output vector can be represented by Formula (2).


$$X={\left(X_{1},X_{2},\cdots X_{m}\right)}^{T}$$ (1)

$$Y={\left(Y_{1},Y_{2},\cdots Y_{p}\right)}^{T}$$ (2)

From this, it can be concluded that $\left(X_{j},Y_{j}\right)$. The input and output vectors of the corresponding jth decision-making unit. This study adopts a CCR model based on the super efficiency DEA model to calculate the efficiency value of technology finance. This study used DEAP 2.1 software to calculate the efficiency of science and technology finance in 30 provinces, cities, and autonomous regions of China in the past 10 years. The results are shown in Table 3.

**Table 3:** Efficiency values of science and technology finance in 30 provinces of China from 2012 to 2021.

AREA	2012	2013	2014	2015	2016	2017	2018	2019	2020	2021
**Beijing**	2.021	2.401	2.613	3.318	4.220	5.184	5.721	5.792	5.765	5.800
**Tianjin**	1.790	1.947	2.072	2.412	2.662	3.312	4.216	4.521	4.852	5.012
**Hebei**	1.347	1.357	1.491	1.552	1.848	2.127	2.537	2.652	2.862	3.016
**Shanxi**	1.151	1.154	1.214	1.343	1.412	2.206	2.892	2.984	3.225	3.684
**Inner Mongolia**	0.911	1.064	1.189	1.200	1.493	1.625	1.855	1.952	2.105	2.632
**Liaoning**	1.076	1.146	1.186	1.212	1.239	2.046	2.577	2.895	3.104	3.584
**Jinlin**	1.601	1.655	1.698	2.056	2.274	2.514	2.946	3.522	3.852	4.125
**Heilongjiang**	1.918	2.106	2.642	2.716	3.155	3.345	4.012	4.212	4.563	4.963
**Shanghai**	2.942	3.515	3.702	4.013	4.477	4.774	5.231	5.365	5.685	5.981
**Jiangsu**	1.951	2.315	2.814	3.422	3.794	4.016	4.315	4.561	4.984	5.257
**Zhejiang**	3.879	4.015	4.116	4.222	4.294	4.313	4.713	4.856	5.352	5.654
**Anhui**	1.393	1.438	1.462	1.556	1.590	1.749	2.057	2.254	2.451	2.698
**Fujian**	2.134	2.652	3.018	3.394	3.851	4.513	4.722	4.951	5.120	4.239
**Jiangxi**	1.452	1.642	1.842	2.312	2.974	3.841	4.532	4.752	4.963	5.120
**Shandong**	1.912	2.310	2.292	2.462	2.755	3.012	3.325	3.658	3.852	4.126
**Henan**	1.302	1.415	1.349	1.642	2.105	2.742	3.215	3.425	3.623	3.891
**Hubei**	1.620	1.783	1.905	2.113	2.709	3.238	3.726	3.982	4.256	4.330
**Hunan**	1.634	1.846	2.310	2.850	2.984	3.210	3.384	3.562	3.985	4.110
**Guangdong**	3.046	3.211	3.491	4.041	4.215	4.419	4.421	4.558	4.952	5.352
**Guangxi**	1.264	1.364	1.549	1.947	2.432	2.963	3.384	3.563	3.862	4.211
**Hainan**	1.760	2.013	2.319	2.538	2.749	2.842	3.216	3.566	3.852	3.958
**Chongqing**	1.984	2.355	2.842	3.216	3.846	4.316	4.763	4.952	5.235	5.456
**Sichuan**	2.776	2.980	3.351	3.475	4.454	4.782	4.579	4.652	4.820	4.963
**Guizhou**	2.516	2.745	2.916	3.412	4.268	4.987	4.859	4.920	5.201	5.520
**Yunnan**	1.459	1.643	1.846	2.125	2.642	3.019	3.416	3.652	3.854	3.971
**Shaanxi**	1.649	1.846	1.994	2.346	2.895	3.459	3.867	3.952	4.156	4.336
**Gansu**	1.945	2.364	2.216	2.542	2.748	2.954	3.515	3.822	4.120	4.522
**Qinghai**	1.245	1.225	1.235	1.469	1.948	2.242	2.689	2.986	3.350	3.963
**Ningxia**	1.875	1.546	1.759	1.932	2.351	2.546	2.754	3.022	3.854	3.741
**Mean**	1.826	2.036	2.239	2.556	2.995	3.420	3.780	3.921	4.202	4.421

#### Measurement of regional real economy indicators

Currently, there is no universally accepted definition of the real economy; however, numerous studies have been conducted in this area. This study proposes that the real economy encompasses economic activities facilitated through social capital. Specifically, it includes direct economic activities associated with material and financial production as well as cultural service consumption. Considering the volatility of China’s real estate market, the regional real economy discussed in this study refers to the residual portion obtained after subtracting the added value contributed by both the real estate industry and financial sector. The growth of regional real economy is defined as actual GDP minus the added value from both real estate and financial industries, with this remaining portion serving as a dependent variable. Regional indicators for real economic growth are calculated based on actual regional GDP and nominal GDP.

#### Measurement of environmental regulation

The efficiency of technology finance in relation to capital flows may be influenced by environmental regulations, which can vary depending on market volatility and subsequently impact regional real economic growth. Therefore, this study considers environmental regulation as a key explanatory variable, specifically focusing on the intensity of such regulation. The intensity is measured by the ratio of regional pollution control investment to regional GDP.

### Analysis of the impact of technology and financial efficiency on regional real economic growth

#### Construction of mediation effect model

**Fig 1:**
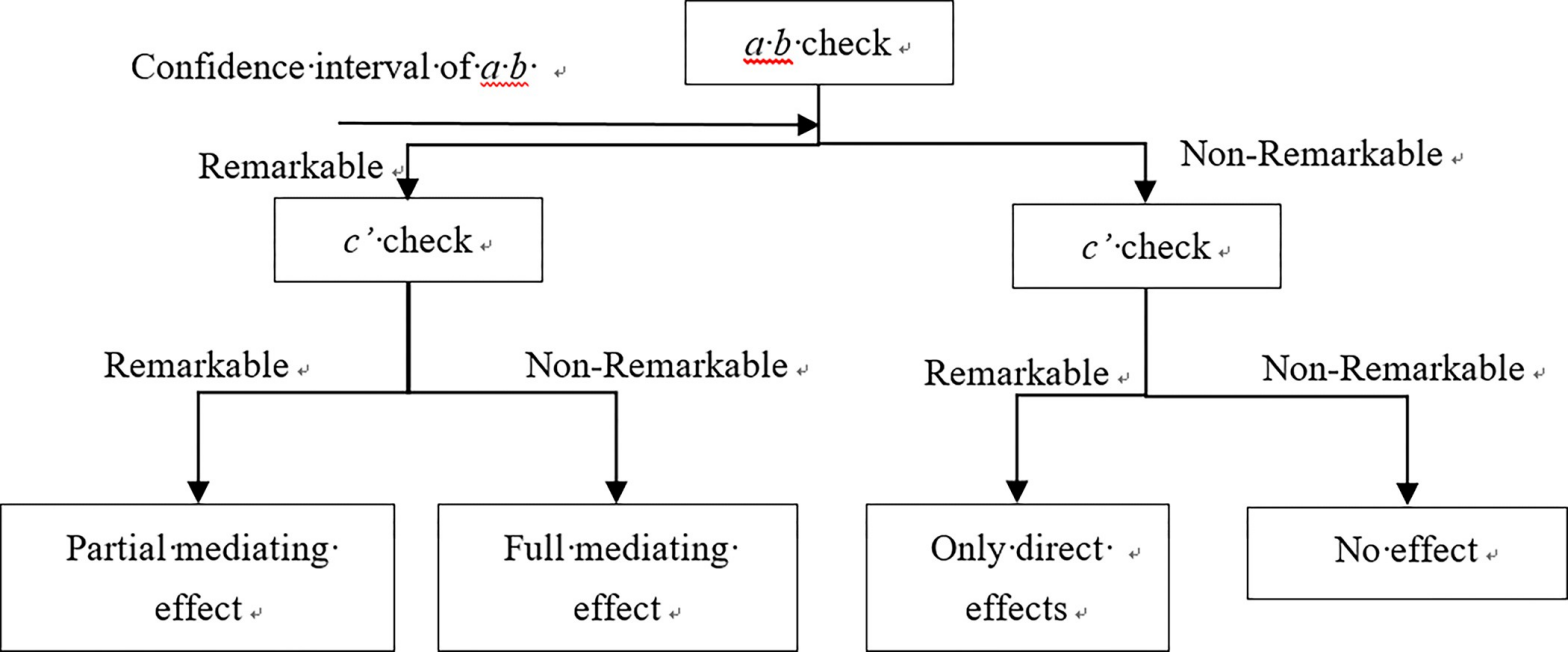
Steps for bootstrap mediation effect test.

Hierarchical regression analysis is a widely employed approach for examining mediating effects. By assessing the significance of regression coefficients from independent variables to dependent variables, independent variables to intermediate variables, and intermediate variables to dependent variables, progression to the next step is contingent upon each test being statistically significant. The presence of statistical significance across all steps indicates a significant mediating effect. In cases where non-significant findings arise during testing, subsequent evaluation using Bootstrap or Sobel tests is conducted to further ascertain the significance of the mediating effect [30]. The specific method steps are shown in Fig 1.

In the above figure, the first step, verify a × b significant (Bootstrap method), if a × b is significant, and the intermediate test result does not include 0 at the 95% confidence interval. If the intermediate path exists, perform the first step of the test. Conversely. If a × b is not significant, then the intermediate test result contains 0 at the 95% confidence interval, and the intermediate path does not exist, skip to the third step;

The second step is to test whether c’ is significant. If c’ is not significant, it is a complete mediating effect. If c’ is significant, it is a partial mediating effect;

Step 3, if a × b is not significant, then the mediation is not established. Continue to test whether c’ is significant. If c’ is significant, then there is only a direct effect. If c’ is not significant, then it has no effect.

In this study, the direct and intermediate effects were tested using the hierarchical regression method, and the regression equation constructed is shown in Formulas (3)–(5).


Y=cX+e1 (3)

Me=aX+e2 (4)

Y=c’X+bMe+e3 (5)

Among them, coefficient c refers to the total effect of the independent variable on the dependent variable, that is, the total effect of the efficiency of technology and finance on the growth of regional real economy. The coefficient a refers to the effect of the independent variable on the environmental regulation of the intermediary variable; The coefficient b refers to the effect of the mediating variable environmental regulation on the regional real economic growth of the dependent variable after controlling for the influence of the independent variable; The coefficient c’ refers to the direct effect of technology and finance efficiency on the growth of regional real economy after controlling for the influence of intermediary variables. e1—e_3_ refers to the regression residual. If there is a mediating effect in the model, then the mediating effect is a × b, so the total effect is c = c’ + a × b.

In the mediation effect test, considering the impact of factors such as urbanization level (Urban), degree of openness to the outside world (Open), and fiscal expenditure (Fis) on environmental regulation intensity and regional real economic growth in the survey sample, the above variables are used as control variables. Among them, Urban refers to the proportion of urban population in total labor force; Open refers to the proportion of the total import and export volume of each region to GDP; Fis refers to the proportion of the total fiscal expenditure of each province to GDP. The data source is the “China Statistical Yearbook” and “Provincial Statistical Yearbook” from 2012 to 2021, and relevant adjustments are made based on local price levels.

#### Robustness test

As for the robustness of this model analysis, the Tobit model is selected for testing in this study. Its principle and hypothesis are shown in the following Eqs (6) and (7).


$$Y^{*}=X\beta +\varepsilon$$ (6)

$$Y2=\max(0,\;Y^{*})=\{\begin{array}{cc} Y^{*} & Y^{*}>0\\ 0 & Y^{*}\leq 0 \end{array}$$ (7)

In the above formula, Y2 represents the level of regional real economy, defined by large, medium and small, and refers to the observable explained variables. At that time, the value of Y2 was 0, and the value of Y2 was the same. Refers to a series of explanatory variables, refers to the random disturbance term, meets the requirements of normal distribution, as shown in Eq (8):

$$\begin{array}{l} Y_{2}=\alpha +\beta_{1}TE+\beta_{2}ERI+\beta_{3}Urban+\beta_{4}Open+\\ \beta_{5}\mathrm{Fis}+\beta_{6}X_{i}+\varepsilon \end{array}$$ (8)

Multiple regression model was used to replace the Tobit model and the results were shown in Table 4.

**Table 4:** Robustness test.

	Probit	Ols
TE	-0.014	-0.065
	(0.021)	(0.015)
ERI	0.078***	0.172***
	(0.025)	(0.022)
Urban	0.047***	0.022***
	(0.016)	(0.013)
Open	-0.027	-0.078
	(0.015)	(0.021)
Fis	-0.036**	-0.122**
	(0.014)	(0.054)

As can be seen from Table 4, after replacing the model, the contribution degree and influence direction of the above five independent variables to the dependent variables did not change significantly. Among the control variables, the influence of Fis is very significant. Even after replacing the model, the influence of the core explanatory variables on the explained variables in this study did not change significantly, and only the correlation coefficient changed. Therefore, the model estimation adopted in this study was robust.

#### Analysis of empirical results

Based on the financial efficiency of science and technology index TE, environmental regulation intensity ERI, and real economy development index Y measured in the previous text, the mediation effect model is used to analyze the mediation effect between the three variables. This study analyzed the mediating effect of optimizing environmental regulations on the efficiency of technology finance in 30 provinces of China on regional real economic growth, and tested the experimental results. Considering the potential regional heterogeneity in technology finance, environmental regulation, and real economy development, this study divides 30 provinces into three regions: Eastern, central, and western. Conduct an empirical analysis on the mediating effects of technology and finance efficiency, environmental regulation intensity, and regional actual economic growth in three regions.

Firstly, conduct a regression analysis on the efficiency of technology finance and regional real economic growth, and obtain a regression coefficient c to test the significance between the two; Secondly, regression analysis is conducted on the impact of technology and finance efficiency on the regional real economic growth as an intermediary variable, and a regression coefficient a is obtained to test the significance between the two; Finally, a hierarchical regression analysis is conducted on the effects of technological and financial efficiency and environmental regulation as mediators on regional real economic growth, from which the regression coefficients are obtained *b* and *c’*. To test the significance between the three variables. The results are shown in Tables 5–7.

**Table 5:** Mediation effect analysis (eastern).

	Y	ERI	Y
**Constant**	-0.625**	1.642**	-1.067**
	(-3.145)	(7.559)	(-5.396)
**Urban**	0.119*	-0.036	0.111*
	(2.419)	(-0.659)	(2.355)
**Open**	0.421**	0.144**	0.399**
	(8.719)	(2.727)	(8.588)
**Fis**	0.021	-0.027	0.029
	(0.836)	(-0.975)	(1.209)
**TE**	0.325**	0.168**	0.154**
	(8.063)	(5.510)	(5.540)
**ERI**			0.229**
			(8.281)
**Sample**	120	120	120
***R* 2**	0.473	0.232	0.518
**Adjust *R* 2**	0.467	0.223	0.510
***F***	*F* (13,1033) = 71.416, *p* = 0.000	*F* (13,1033) = 24.036, *p* = 0.000	*F* (16,1030) = 69.143,*p* = 0.000

Note: * p<0.05

* * p<0.01. All variables in the table are brought into the regression equation using the mean (t-value in parentheses) (the same below).

**Table 6:** Mediation effect analysis (central).

	Y	ERI	Y
**Constant**	1.347**	0.730**	0.172**
	(6.407)	(3.408)	(5.699)
**Urban**	0.022	0.049	0.263**
	(0.429)	(0.929)	(8.866)
**Open**	0.014	0.041	0.278**
	(0.274)	(0.793)	(9.538)
**Fis**	-0.046	-0.000	0.192**
	(-1.720)	(-0.018)	(6.502)
**TE**	0.313**	0.172**	0.257**
	(7.223)	(5.699)	(8.824)
**ERI**			0.322**
			(11.885)
**Sample**	90	90	90
***R* 2**	0.453	0.231	0.448
**Adjust *R* 2**	0.457	0.253	0.515
***F***	*F* (13,1033) = 35.204, *p* = 0.000	*F* (13,1033) = 37.392, *p* = 0.000	*F* (16,1030) = 71.232,*p* = 0.000

**Table 7:** Analysis of mediation effects (western region).

	Y	ERI	Y
**Constant**	1.347**	0.730**	0.257**
	(6.407)	(3.408)	(8.824)
**Urban**	0.022	0.049	0.263**
	(0.429)	(0.929)	(8.866)
**Open**	0.014	0.041	0.192**
	(0.274)	(0.793)	(6.502)
**Fis**	-0.046	-0.000	0.213**
	(-1.720)	(-0.018)	(7.223)
**TE**	0.209**	0.211**	0.213**
	(11.258)	(7.036)	(7.579)
**ERI**			0.222**
			(7.756)
**Sample**	90	90	90
***R* 2**	0.474	0.433	0.508
**Adjust *R* 2**	0.468	0.228	0.511
***F***	*F* (13,1033) = 45.194, *p* = 0.000	*F* (13,1033) = 36.352, *p* = 0.000	*F* (16,1030) = 58.154,*p* = 0.000

From the data in the eastern part of Table 5, it can be seen that the efficiency of technology finance has a significant positive predictive effect on regional real economic growth (c = 0.325); The efficiency of technology finance also has a significant positive predictive effect on environmental regulation (a = 0.168); The mediating variable environmental regulation also has a significant positive predictive effect on regional real economic growth (b = 0.222); When environmental regulations are included as mediating variables in the model, the positive predictive effect of financial efficiency of science and technology on regional real economic growth becomes less significant (c’ = 0.213).

Similarly, in the central data of Table 6, it can be seen that the efficiency of technology finance has a significant positive predictive effect on regional real economic growth (c = 0.313); The efficiency of technology finance also has a significant positive predictive effect on environmental regulation (a = 0.172); The mediating variable environmental regulation also has a significant positive predictive effect on regional real economic growth (b = 0.322); When environmental regulations are included as mediating variables in the model, the positive predictive effect of financial efficiency of science and technology on regional real economic growth becomes less significant (c’ = 0.257).

From the western data in Table 7, it can be seen that the efficiency of technology finance has a significant positive predictive effect on regional real economic growth (c = 0.209); The efficiency of technology finance also has a significant positive predictive effect on environmental regulation (a = 0.211); The mediating variable environmental regulation also has a significant positive predictive effect on regional real economic growth (b = 0.222); When environmental regulations are included as mediating variables in the model, the positive predictive effect of financial efficiency of science and technology on regional real economic growth becomes less significant (c’ = 0.213).

On the previous text, c represents the regression coefficient between X and Y (when there is no intermediate variable M in the model), which is the total effect; A represents the regression coefficient between X and M, b represents the regression coefficient between M and Y, and a × b is the product of a and b, which is the mediating effect; 95% Boot CI represents the 95% confidence interval obtained from Bootstrap sampling calculation. If the interval does not include 0, it indicates significance; C’ represents the regression coefficient between X and Y (when there is an intermediate variable M in the model), which is the direct effect; If a and b are significant, and c ‘is not significant, then it is a complete mediator; If a and b are significant, and c ‘is significant, and a × b is the same sign as c’, then it is a partial mediating effect;

If at least one of a and b is not significant, and the 95% Boot CI of a × b includes the number 0 (not significant), then the mediating effect is not significant. If at least one of a and b is not significant, and the 95% Boot CI of a × b does not include the number 0 (significant), and c ‘is not significant, then it is a complete mediator; If at least one of a and b is not significant, and the 95% Boot CI of a × b does not include the number 0 (significant), and c’ is significant, and a * b is signed with c’, then it is a partial mediating effect.

Based on this, the mediating effect of this study was tested, and the results are shown in Table 8.

**Table 8:** Summary of mediation test results.

Term	c	a	b	a × b	a × b (Boot SE)	a × b (z)	a × b (p)	a × b (95% Boot CI)	c’	Inspection conclusion
**Eastern region: financial efficiency of science and technology = >Environmental regulation = >Regional real economy growth**	0.325**	0.168**	0.222**	0.016	0.006	2.591	0.010	0.004–0.027	0.154**	partial mediation
**Central region: financial efficiency of science and technology = >Environmental regulation = >Regional real economy growth**	0.313**	0.172**	0.322**	0.026	0.007	3.507	0.000	0.010–0.040	0.154**	partial mediation
**Western region: financial efficiency of science and technology = >Environmental regulation = >Regional real economy growth**	0.209**	0.211**	0.222**	0.020	0.007	2.651	0.008	0.006–0.036	0.213**	partial mediation

From the above table, it can be seen that the efficiency of technology and finance in the eastern, central, and western regions has a significant and documented positive impact on regional real economic growth. The efficiency of technology and finance has a significant positive impact on environmental regulation, and environmental regulation also has a significant positive impact on regional real economic growth. This indicates that the mediating effect of technology finance on regional real economy growth through environmental regulation in various regions is significant, and the mediating effect in the eastern region is greater than that in the western region. There are regional differences in the mediating effect of technology finance on regional real economy growth through environmental regulation.

Similarly, the mediating effect of this study was tested, and the results are shown in Table 9.

**Table 9:** Summary of mediation test results.

Term	c	a	b	a × b	a × b (Boot SE)	a × b (*z*)	a × b (*p*)	a × b (95% Boot CI)	c’	Inspection conclusion
**financial efficiency of science and technology = >Environmental**	0.862**	0.663**	0.390**	0.258	0.022	11.532	0.000	0.147–0.232	0.604**	partial mediation

* *p*<0.05

** *p*<0.01

In summary, it is not difficult to see that environmental regulations play a partial intermediary role between the efficiency of technology finance and the growth of regional real economy. That is to say, the efficiency of technology finance has a significant positive effect on environmental regulation, and environmental regulation has a significant positive effect on regional real economic growth. Therefore, environmental regulation has a mediating effect between financial efficiency of science and technology and regional real economic growth. However, the efficiency of technology finance also plays a significant positive role in the growth of regional real economy, which makes the mediating role played by environmental regulations not entirely mediating, but partially mediating. The relationship between the efficiency of technology finance and the growth of regional real economy is not only influenced by environmental regulations, but also by many other factors. For example, it is influenced by factors such as Uran, Open, Fi, and industrial structure [18]. This to some extent weakens the chain effect of financial efficiency of science and technology and environmental regulation on regional real economic growth, resulting in a direct relationship between financial efficiency of science and technology and regional real economic growth, with environmental regulation playing a partial intermediary role between the two.

## Conclusion

Based on the current situation of science and technology finance, environmental regulation, and regional real economy development in China, this paper analyzes the theoretical relationship among these three factors. It then utilizes data from 2012 to 2021 based on 2011 to construct an index system for each factor, measure their respective indices, and build an intermediary effect model. This study examines the mediating effect of environmental regulation intensity on the influence of science and technology financial efficiency on real economic growth. Additionally, it investigates the mediating effect of industrial structure optimization on the impact of science and technology financial efficiency on real economic growth in three regions. The research findings are as follows: (1) The enhancement of science and technology financial efficiency has a significant positive impact on China’s regional real economy growth. Over the past decade, there has been a general increase in science and technology financial efficiency across China’s 30 provinces and autonomous regions; however, there exists an imbalance in its development between central/western regions compared to other areas. (2) Science and technology financial efficiency significantly affects environmental regulation intensity, with environmental regulation playing a crucial intermediary role in linking science and financial efficiency of science and technology to real economic growth. (3) There are regional disparities regarding the intermediary effect of science and technology finance on real economic growth through increased environmental regulation intensity; specifically, this effect is more pronounced in eastern regions compared to central or western ones. Therefore, enhancing both the development and efficacy of sci-tech finance is essential for promoting real economic growth.

In order to further promote science and technology finance to empower regional real economic growth, policy recommendations that can be adopted:

1. We will strengthen financial support for local governments. Chinese government departments should be able to formulate highly targeted legal provisions to effectively implement fiscal and tax policies into specific green spending practices such as energy conservation, pollution reduction, and carbon reduction.
2. Increase the intensity of fiscal investment in science and technology, and establish a mechanism for the growth of fiscal green spending on science and technology. Strengthen the role of government green guidance funds, vigorously develop government guidance funds, and increase the amount of venture capital for regional real economy enterprises.
3. Optimize the government-guided venture capital mechanism. Through the establishment of special channels, improve the level of internal management, and increase the proportion of total government investment.
4. Vigorously support and build a science and technology financial service platform, and formulate a coordinated development strategy for the coupling of environmental regulation and science and technology finance according to different local economic development levels and ecological civilization construction requirements.
5. The central and western regions should give priority to the development of science and technology finance, and strengthen the research and development institutions within leading financial institutions and large industrial enterprises.
